# Intronization, de-intronization and intron sliding are rare in *Cryptococcus*

**DOI:** 10.1186/1471-2148-9-192

**Published:** 2009-08-07

**Authors:** Scott W Roy

**Affiliations:** 1 National Center for Biotechnology Information, National Library of Medicine, National Institutes of Health, Bethesda, MD, USA

## Abstract

**Background:**

Eukaryotic pre-mRNA gene transcripts are processed by the spliceosome to remove portions of the transcript, called spliceosomal introns. The spliceosome recognizes intron boundaries by the presence of sequence signals (motifs) contained in the actual transcript, thus sequence changes in the genome that affect existing splicing signals or create new signals may lead to changes in transcript splicing patterns. Such changes may lead to previously excluded (intronic) transcript regions being included (exonic) or vice versa. Such changes can affect the encoded protein sequence and/or post-transcriptional regulation, and are thus a potentially important source of genomic and phenotypic novelty. Two recent papers suggest that such changes may be a major force in remodeling of eukaryotic gene structures, however the rate of occurrence of such changes has not been assessed at the genomic level.

**Results:**

I studied four closely related species of *Cryptoccocus *fungi. Among 28,256 studied introns, canonical GT/C...AG boundaries are nearly universally conserved across all four species. Among only 40 observed cases of cDNA-confirmed non-conserved intron boundaries, most are likely to involve alternative splicing. I find only five cases of "intronization," intron creation from an internal exonic region by de novo emergence of new splicing boundaries, and no cases of the reverse process, "de-intronization." I find no more than ten clear cases of true movement of an intron boundary of a possibly constitutively spliced intron, and no clear cases of true "intron sliding," in which changes in the positions of both intron boundaries could lead to a movement of the intron position along the coding sequence.

**Conclusion:**

These results suggest that intronization, de-intronization, and intron boundary movement are rare events in evolution.

## Background

The spliceosome is a large RNA-protein complex that processes pre-mRNA transcripts to remove some regions of transcripts, called spliceosomal introns (hereafter simply 'introns'; included regions are called 'exons'). Some genes exhibit alternative splicing, with some genic regions being included in some but not all mRNA transcripts [1]. Intron-exon structures also change through evolutionary time [2]. Changes can occur either by gain or loss of a large amount of genomic sequence (for instance by complete or nearly complete loss of one or more complete intron or exon; [3-8]), or by the evolution of new splicing patterns for existing sequence due to small-scale sequence changes ([9,10]; more complex cases are also possible).

Intron-exon structures can change due to large-scale gain/loss of sequence in several ways. Precise loss of an intronic sequence can occur by genomic recombination with a reverse transcribed mRNA [3-5]. Introns or exons can also be lost by exact or inexact genomic deletion, leading to loss of splicing in that region [11,12]. Exons, and conceivably introns, can arise by insertion of a transposable element or by tandem duplication of a genic region [10,13-17].

Smaller-scale sequence changes may also lead to changes in intron-exon structure, with formerly exonic sequence becoming intronic or vice-versa. Transcript splicing patterns are determined by generally short sequence motifs, including intron boundary motifs (minimally a 5' GT/C and a 3' AG) and an internal 'branch point' motif which plays a key role in the splicing reaction (typically TNA, where N is any nucleotide), as well as various more diffuse intronic and exonic sequence signals [18]. As such, small-scale mutations may either disrupt splicing signals or create cryptic splicing signals, leading to changes in splicing patterns, converting formerly intronic sequence into exonic sequence or vice versa [19-22].

Such sequence changes can increase or decrease the total number of introns/exons in the gene, or simply change the position of an existing splicing boundary. Intron/exon number can increase by emergence of new splice boundaries, either (i) within an intron, leading to 'exonization' of an internal portion of an intron (studied extensively previously, see for instance reference 12); or (ii) within an exon, leading to 'intronization' of an internal portion of an exon [9,23]. Intron/exon number can decrease by (i) loss of splicing of an intron ('de-intronization,' converting an intron into an internal portion of an exon; [9]); or (ii) loss of splicing of an exon ('de-exonization,' converting an exon into an internal portion of an intron). Alternatively, specific sequence changes can affect boundaries of existing introns, leading to movement of intron-exon boundaries along the sequence, converting sequence near intron-exon boundaries from exonic to intronic, or vice versa.

Recently, two sets of authors suggested that sequence changes leading to changes in splicing patterns could be important forces in the evolutionary remodeling of eukaryotic gene structures. First, Tarrio-Rodriguez et al. [22] presented a model by which the boundaries of an intron could migrate along a gene by compensatory changes at both ends of the intron, with alternative use of ancestral and new boundaries providing a viable evolutionary intermediate. They suggested that such 'intron sliding' events could be a major contributor to the observed diversity of intron positions among eukaryotic homologs [2,4,6,7]. Second, Catania and Lynch [23] argued that intronization could be a major force in the shaping of gene structures. However, the actual evolutionary rates at which intronic sequence becomes exonic and vice versa remain unknown.

**Figure 1:**
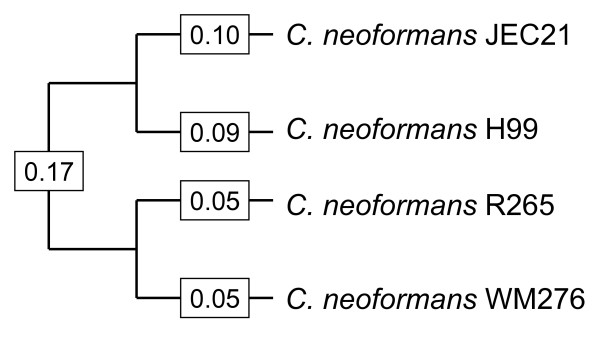
**Species studied, with estimated branch length, in average changes per synonymous site (dS), from reference **[28].

In order to understand tempo and mode of evolution of intron boundaries, I studied four-species genomic alignments of *Cryptococcus neoformans *species (Figure 1; [24]). Unlike ascomycetous fungi, *Cryptococcus *genes are very intron-rich, and show considerable alternative splicing [25,26], and thus are of interest in comparing and contrasting with the better-studied intron-rich genomes of animals and plants. Several studies focusing on these species have elucidated the evolution of intron-exon structures in this group. It has previously been shown that intron loss and gain by insertion/deletion of a complete or near-complete intronic sequence from the genome is a very rare event among these species [27,28]. Myself and others studied introns in the untranslated regions of transcripts, and found a high level of conservation of splicing boundaries both upsteam and downstream of the coding region, indicating the action of purifying selection in maintaining splicing in non-coding regions [29]. Hughes et al. studied indels within *Cryptococcus *introns, and reported evidence for selection driving introns towards an optimum length [24]. Other insights come from more phylogenetically broad many-species studies. Warnecke et al. showed skewed amino acid usage near intron-exon boundaries in *Cryptococcus*, suggesting that splicing in *Cryptococcus*, as in animals and plants, makes use of so-called exonic splicing enhancers and exonic splicing silencers. In their study of transcript variation across eukaryotes, McGuire et al. [26] showed that alternative splicing in *Cryptococcus *is dominated by intron-retention events (in which the entire sequence that is spliced out of one transcript of a gene is entirely retained in another transcript).

I studied genomic regions corresponding to cDNA-confirmed introns in the reference species JEC21, previously studied by Hughes et al. [24]. Intron boundaries are almost universally conserved, with the GT..AG intron boundaries retained across all four species for 99.9% of introns studied. Among the 40 introns for which boundaries are not conserved, only ten are likely cases of change of a constitutive boundary; other cases are consistent with alternative splicing. I find only five apparent cases of intronization, and no cases of de-intronization. Thus changes of intron-exon structures by small-scale sequence changes may be rare events in evolution.

## Results

### Conservation of intron boundaries in Cryptococcus

I studied 28,256 predicted introns and flanking exonic sequences in the reference species JEC21, and the corresponding regions from three other *Cryptococcus *species (Figure 1; [24]). I found only 40 cDNA-confirmed introns in which GT/C...AG boundaries were not preserved in the corresponding position across all species (see Methods). Thus, roughly 99.9% of intron boundaries examined here are conserved across the entire clade.

### Introns with boundary changes

**Figure 2:**
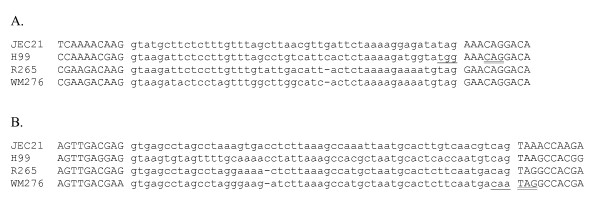
**Most cases of non-conserved boundaries have nearby in frame candidate alternative boundaries**. Lower/upper case sequence indicates intronic/exonic sequence as defined for JEC21. A. Intron 1 of gene CND03550. The JEC21 TAG 3' intron boundary is mutated to TGG in H99 (single underline). H99 may use the downstream CAG instead (double underline). B. Intron 1 of gene CNC04760. The JEC21 CAG 3' intron boundary is mutated to CAA in WM276 (single underline). WM276 may use the downstream TAG instead (double underline). In all cases, intron numbers are given in reference to the coding sequence.

The 40 observed exceptions are summarized in Table 1. In 24/40 cases, the non-conserved JEC21 intron boundary falls within 9 bp of a possible alternative in frame boundary (that is, an GT or AG), such that each species has at least one potential splicing boundary (Figure 2). In 14/24 of these cases, the alternative boundary is conserved across all four species (e.g., Figure 2a). This pattern is consistent with usage of alternative boundaries in JEC21 (supported by cDNA evidence for usage of alternative boundaries in 4 cases), and with usage of only a single boundary in some relatives. Given the large number of cases examined, and the small number of available ESTs per gene, it remains possible that the evolutionarily conserved boundary is in fact the more commonly used splice site in JEC21 as well.

**Table 1:** A summary of the 40 observed exceptions

		**Non-**	**Alternative**	**JEC21/alt boundary**					
	**Int**	**conserved**	**In Frame**	**Presence/Absence**				**Frame**	
**Gene**	**#**	**Boundary**	**Boundaries**	**JEC21**	**H99**	**R265**	**WM276**	**shift**	**Comments**
Conserved alternative boundary									
CNA00590	1	5'	-6 (GTGAGT)	+/+	+/+	-/+	-/+	Yes	cDNA supporting alternative boundary
CNA03150	3	3'	-9 (CAG)	+/+	-/+	-/+	-/+	Yes	
CNC00370	9	5'	+3 (GTGAGT)	+/+	-/+	-/+	-/+	Yes	cDNA supporting intron ret.
CNC01260	8	3'	+6 (TAG)	+/+	+/+	-/+	-/+	Yes	cDNA supporting alt boundary, intron ret.
CND02500	1	3'	+6 (TAG)	+/+	+/+	-/+	-/+	Yes	cDNA supporting out-of-frame boundary
CND03170	8	5'	+6 (GTAAGT)	+/+	+/+	-/+	+/+	Yes	
CND03550	1	3'	+6 (CAG)	+/+	-/+	+/+	+/+	Yes	Fig 2A
CND03880	7	3'	-3 (CAG)	+/+	+/+	-/+	-/+	Yes	
CND04350	1	3'	+9 (C/TAG)	+/+	-/+	-/+	-/+	Yes	
CNE00380	3	3'	+3 (C/AAG)	+/+	-/+	-/+	-/+	No	cDNA supporting alternative boundary
CNE01370	3	3'	+9 (CAG)	+/+	+/+	+/+	-/+	Yes	
CNF02810	9	3'	+9 (C/TAG)	+/+	+/+	-/+	-/+	Yes	
CNH03260	8	3'	+6 (TAG)	+/+	-/+	-/+	-/+	Yes	
CNK01580	2	3'	+6 (C/TAG)	+/+	+/+	-/+	-/+	No	cDNAs supporting alternative boundary
Non-conserved alternative boundaries									
CNA01650	1	3'	-3 (TAG)	+/-	-/+	-/+	-/+	Yes	
CNB04950	6	5'	+3 (GTTCAC)	+/-	+/-	-/+	-/+	No	
CNC04760	1	3'	+3 (TAG)	+/-	+/-	+/+	-/+	Yes	Fig 2B
CND00770	1	3'	-3 (CAG)	+/-	+/-	+/-	-/+	Yes	
CNH03490	7	3'	-12 (CAG)	+/-	+/-	-/+	-/+	Yes	
CNI02860	6	5'	+3 (GTTCAT)	+/-	+/-	+/-	-/+	Yes	
CNK00690	2	3'	+6 (CAG)	+/+	-/+	+/-	+/+	Yes	
CNL04670	4	3'	+9 (C/TAG)	+/-	+/+	-/+	-/+	Yes	
CNL06040	1	3'	-3 (GAG)	+/-	+/-	+/+	-/+	Yes	
CNM02250	2	3'	-9 (AAG)	+/-	+/-	-/+	-/+	No	
Intronization/de-intronization									
CND01950	3	3'	No	+/	-/	-/	-/	No	Intronization Fig 3B (1/0 cDNAs spliced/unspliced)
CNF02770	6	Both	No	+/	-/	-/	-/	No	Intronization Fig 3E (1/7 cDNAs spliced/unspliced)
CNG00990	3	Both	No	+/	-/	-/	-/	No	Intronization Fig 3A (1/4 cDNAs spliced/unspliced)
CNL03830	4	3'	No	+/	-/	-/	-/	No	Intronization Fig 3D (1/7 cDNAs spliced/unspliced)
CNI02050	4	Both	No	+/	-/	-/	-/	No	Intronization Fig 3C (2/4 cDNAs spliced/unspliced)
CNL04380	1	3'	No	+/	+/	-/	-/	Yes	Direction unclear Fig 4A (1/0 cDNAs spliced/unspliced)
CND00470	1	5'	No	+/	-/	-/	+/	No	Convergent Fig 4B (2/0 cDNAs spliced/unspliced)
More complex cases									
CNB05120	2	5'	No	+/	+/	-/	+/	Yes	Unclear (53 bp)
CNF04220	1	Both	No	+/	+/	-/	-/	Yes	Unclear (58 bp)
CNI04240	1	3'	No	+/	-/	+/	+/	Yes	Unclear (64 bp)
CNJ02980	2	5'	No	+/	+/	-/	-/	Yes	Unclear (52/53 bp)
CNK02560	2	3'	No	+/	-/	-/	-/	Yes	Unclear (136/137/143 bp)
CNK02690	2	3'	No	+/	+/	-/	-/	Yes	Unclear (57/58 bp)
CNL06330	4	3'	No	+/	-/	+/	+/	Yes	Unclear (62 bp)
CNM00170	5	Both	No	+/	-/	-/	-/	Yes	Unclear (Fig 4C), unspliced cDNA (61/64 bp)
CNN00940	2	3'	No	+/	-/	+/	+/	Yes	Unclear (84 bp, inframe stop)

In the other ten cases, the in frame candidate alternative boundary is not conserved across all four species, however the sequence appears to be intronic across species: the sequence interrupts the coding frame and/or exhibits indel frameshift mutations between species, strongly suggesting that the region is spliced across all four species (e.g., Figure 2b). These are candidates for true boundary movement, in which different species constitutively utilize different sites for intron splicing.

**Figure 3:**
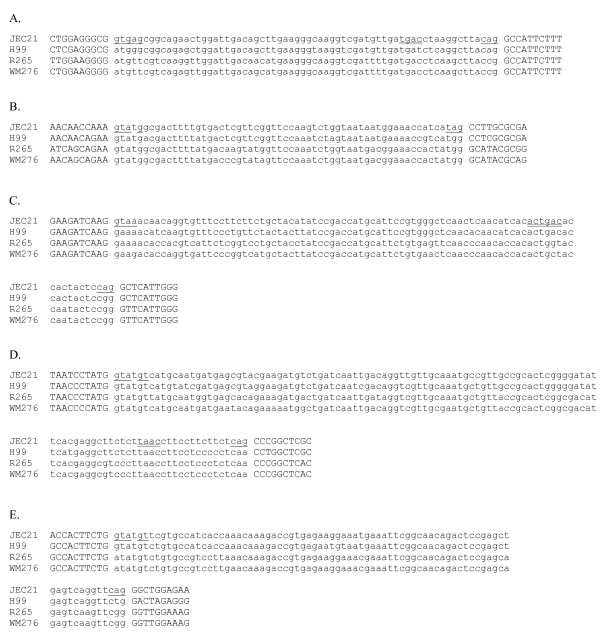
**Apparent cases of intronization of exonic sequence**. In each case, nucleotides conforming to extended consensus sequences (GTRAGT and YAG) are underlined, and branch-point like sites are double underlined. A. Intron 3 of CNG00990; B. Intron 3 of CND01950; C. Intron 5 of CNI02050; D. Intron 4 of CNL03830; E. Intron 6 of CNF02770.

**Figure 4:**
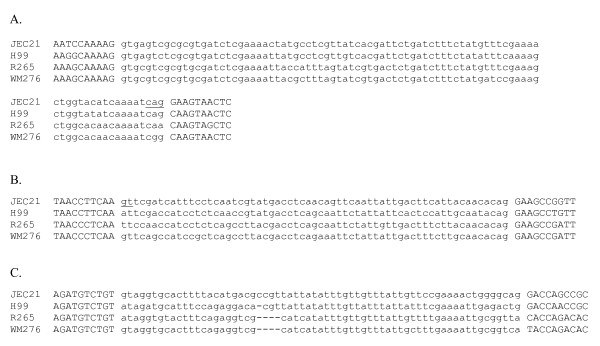
**Complex cases of intron boundary evolution**. A. Intron 5 of CNM00170. Both boundaries of the intron in JEC21 are non-conserved, however the presence of gaps inducing frameshifts suggests that the region is intronic across all species. B. Intron 1 of CND00470. Only WM276 shares the JEC21 5' intron boundary, suggesting either convergent intronization in JEC21 and WM276 or convergent loss of splicing in the other two species. C. Intron 5 of CNM00170. The 5' intron boundary is not conserved, and there is no nearby alternative candidate boundary, however the presence of coding frame-interrupting indels suggests that the sequence is ancestrally intronic.

I also uncovered five apparent examples of 'intronization' [9,23] in which an internal portion of an exon is converted to a new intron by acquisition of splicing boundaries (Figure 3). In each case, only JEC21 exhibits GT...AG splicing boundaries; in 4/5 cases, cDNA evidence indicated that the intronic sequence was alternatively spliced (i.e., 'intron retention' – cDNAs were found both containing and lacking the intronic sequence). Consistent with the ancestral exonic character of the sequence, coding frame and amino acid sequence encoded were highly conserved across species within these regions, and all new introns were a multiple of 3 nts, thus conserving coding frame (Figure 3). In addition, I found two cases with conserved coding frame and amino acid sequence within the spliced region, in which the JEC21 splicing boundaries were shared with one other species. In one case (Figure 4A), H99 also shares the JEC21 splicing boundaries, consistent with either intronization in the H99-JEC21 ancestor, or ancestral alternative splicing and loss of splicing in the R265-WM276 ancestor (de-intronization). In the other case, splicing boundaries are present only in JEC21 and the more distant relative, consistent with either convergent gain or loss of splicing (Figure 4B).

In the remaining cases, the utilized intron boundaries in JEC21 were absent in some of the other three species, without nearby candidates for alternative splicing boundaries, however the high observed degree of evolutionary change within the sequence, generally including frameshift-causing indels, suggested that the sequence was ancestrally intronic. These are possible examples of long-range changes in intron boundaries within the clade. In addition, in some cases long indels overlapping the intron/exon boundaries attested to more profound rearrangements of gene and intron-exon structures. An example of such a complex change are given in Figure 4C.

### No de-intronization

Intronization is the conversion of a single exon into two exons and an intervening intron. What of the reverse process, 'de-intronization,' with loss of intron splicing converting two exons and an intervening intron into a single long exon? (It is worth distinguishing de-intronization from exonization, in which an internal portion of an intron, but not the whole intron, becomes exonic.) Could some of the above 'complex' cases represent de-intronization events? In de-intronization, newly exonic sequence would be expected to respect coding frame – thus in all species without splicing boundaries the corresponding regions would expected to be a multiple of three basepairs and to lack in frame stop codons. This pattern is not found for any of the complex cases (Table 1), thus there are no clear candidates for de-intronization events.

### Features of boundary changes

Among the 40 introns with changed boundaries, five introns exhibited changes at both boundaries, and 35 had changes at only one. 3' changes were much more common, with 83% (29/35) of changes at the 3' boundary (*P *= 5.8 × 10^-5 ^by a binomial test for equal probabilities). In 66% (24/35) of cases, there was a candidate alternative in frame boundary (GT or AG) within 9 nts of the JEC21 boundary position (and one case of an alternative boundary 12 nt away), and many of these alternative boundaries had extended consensus sequences (i.e., a 5' GT/CRAG or 3' YAG; Table 1). The difference in numbers of non-conserved 5' and 3' boundaries may be understood in this context: given the greater information content to 5' boundaries (with an extended GTRAGT consensus), truly alternative 5' boundaries are less likely to occur at random, thus loss of an ancestral boundary is less likely to be compensated by an alternative boundary.

Alternative boundaries were slightly but not significantly more likely to fall downstream of the JEC21 boundary ('+' changes Table 1; 16/25 in all; *P *= 0.12) for both 5' changes (4/5) and 3' changes (12/20). Use of the alternative boundary was observed (i.e. alternative splicing) for 4 out of 15 cases for which multiple spliced cDNA transcripts were available, and an additional out-of-frame 3' boundary was found to have been used in a single cDNA for one additional case. In addition, cDNAs with the intron sequence retained were found in 2/25 cases (Table 1).

I compared the 40 introns with observed boundary sequence changes with 10,000 randomly generated sets of 40 introns chosen from among all JEC21 introns. Introns undergoing changes were longer than expected (only 252 sets had greater or equal mean length: *P *= 0.025), and showed weaker overall adherence to 5' boundary consensus (average number of matches to the GTRAGT consensus was lower than for all but 96 random sets: *P *= 0.096). The difference was not significant when intronic sequence positions were considered separately, except for the sixth position, at which introns undergoing changes were less likely to contain a consensus T than expected, at a *P *= 0.017 level, correcting for multiple comparisons.

## Discussion

### No intron sliding or de-intronization

Intron positions often differ across conserved regions of homologs, attesting to evolutionary plasticity of intron-exon structures. Here I study three possible contributing mechanisms to this remodeling of gene structures: intron sliding [22,30,31], by which an intron may migrate a short distance along a gene, for instance by sequential changes of intron boundaries facilitated by alternative splicing [22]; intronization, conversion of an internal portion of an exon into a new intron); and de-intronization, loss of splicing of an intron. This study is the first to systematically evaluate the incidence of such events among species that are closely related enough to confidently infer sequence changes. I found only five cases of intronization and no cases of intron sliding or de-intronization. The excess of intronization over de-intronization echoes the case in *Caenorhabditis *nematodes found in our previous study [9], and contrasts with the excess of intron loss (by loss of the actual intron sequence) over insertion of new introns in the same species studied here [27].

The lack of de-intronization is striking. In the *Cryptococcus *genome, approximately 6.2% of intron sequences do not interrupt coding frame. These non-frame-interrupting introns have a mean of 63.3 bp, thus loss of splicing of any of these introns would amount to an insertion of 21 codons on average. Using the estimated dS (0.46) as an estimate of the amount of mutation within the history of these species, 0.46 × 4 changes interrupting the canonical GT/C..AG splicing boundaries would be expected per intron in the history of these species, or a total number of splicing interrupting mutations equal to 28,256 introns × 6.2% × 0.46 × 4 = 3235 de-intronization events. While this estimate is clearly quite rough, that no such events are observed indicates nearly universal efficient selection opposing such insertions of moderate length.

### Alternative splicing and the evolution of intron-exon structure

In most cases, non-conserved splicing boundaries lie near clear candidates for alternative splicing boundaries. In some cases cDNAs confirm alternative use of the two boundaries in JEC21. The non-conserved JEC21 boundary may dominate, but the alternative boundary may be used (at some level of efficiency) in the absence of the non-conserved boundary. Indeed, given the low overall level of transcript coverage, the 'alternative' boundary may be most frequently used even in JEC21, and by chance the few available transcript sequences reflect the less frequent boundary, thus loss of frequently-used splicing boundaries may be even less common than this study suggests.

### General applicability of the present results: boundary constraints and population size

Is lack of changes at intron boundaries likely to be common across eukaryotes? Presumably, selection against boundary changes will be inversely related to the availability of true alternative boundaries, and the overall efficacy of selection in general (effective population size). Alternative boundaries are likely to be less frequent in intron-poor species such as *S. cerevisiae*, which require extended splice boundaries (e.g., GTATGT) for splicing, and more frequent in species with more lax splice boundaries, which tend to rely on more distant splicing factors (*Cryptococcus *is somewhat intermediate among eukaryotes in the degree of adherence to the splicing boundary consensus sequence; see references [32,33]).

The case for strength of selection is less straightforward. Recently, Lynch and colleagues have presented a series of arguments for a major role of slightly deleterious events in the evolution of genome structure (e.g., [23,34,35]). Within this paradigm, slightly-deleterious mutations may be tolerated in species with small effective population size, but not in larger populations, since the overall effectiveness of selection is dependent on the difference in fitness multiplied by the effective population size. Thus, boundary changes that are effectively selected against in *Cryptococcus*, which is estimated to have a relatively large effective population size [34,35], might be tolerated (i.e., be able to drift to fixation) in a smaller population.

In discerning the role of effective population size in determining genome structures, it is important to keep in mind that the selective effects on different mutations, even superficially similar ones such as analogous mutations at different intron boundaries, are likely to vary widely. Different intron boundaries do or do not have available alternative splice boundaries, alternative boundaries will have different splicing efficiencies across introns, the impacts of intron boundary shifts on mRNA transcripts and resulting proteins will vary across introns, strength of selection against inefficient splicing is likely to vary across introns, and so forth.

What is notable, then, is that nearly all boundaries in *Cryptococcus *– relatively weakly and relatively strongly selected – are preserved by selection. As any of the above variables seems likely to vary by at least an order of magnitude, overall strength of selection on intron boundaries is likely to vary by multiple orders of magnitude. Thus, while a large decrease in effective population size relative to *Cryptococcus *could lead to some intron boundaries becoming effectively neutral, it would likely require a tremendous change in effective population size to lead to a significant rate of change for a substantial fraction of boundaries.

### Change of intron boundaries through eukaryotic evolution

These results allow us to make rough estimates of the contribution of these processes to modern intron-exon structures. Among the species studied, divergence at synonymous sites is roughly 0.46, and degree of intron boundary change is roughly 10 changes for 28,256 introns, or 0.00077 times the rate of divergence at silent sites, per intron. Rates of divergence at synonymous sites have been estimated for a few divergent eukaryotic lineages, and are typically around 5 × 10^-8 ^to 5 × 10^-9^, per year [36-40]. This would suggest a rate of boundary sliding for *Cryptococcus *introns of around 4 × 10^-11 ^or 4 × 10^-12 ^per year. Thus, over roughly two billion years of eukaryotic evolution, the probability of a given intron in a given lineage having undergone a boundary change would be on the order of 0.008 to 0.08. On the simplest model, the probability of an intron undergoing 'sliding,' by change at both boundaries, would be the square of this probability, or 0.00006 to 0.006 (and the probability of changes of the exact number of nucleotides of the exact number of bases at both boundaries, obscuring the change, would presumably be rarer still). These very rough estimates suggest that intron sliding may not be a major contributor to intron position diversity among homologs.

We can similarly ask about the contribution of intronization to modern genomes. Introns arising from intronization appear to be initially alternatively spliced (i.e., the sequence is either spliced or retained; [9]). Over time, the sequence may either revert to the non-spliced form by losing the new splicing boundaries, or may become a constitutively spliced intron (it is also possible that the alternative spliced form could persist, however such 'intron retention' alternative splicing events are not known to be preserved over long evolutionary times; see reference [9]). If the intronized sequence become constitutively spliced, it is then presumably vulnerable to subsequent intron loss. Thus, the probability that an initial intronization is responsible for an observed modern intron is equal to *c*(1-*L*), where *c *is the probability that the intron becomes constitutive (rather than reverts) and *L *is the probability that it is subsequently lost. Thus the total contribution of intronization events to a modern species would be equal to *I *× *c*(1-*L*), where *I *is the number of initial intronization events occurring in the history of the species (i.e., since the origin of spliceosomal introns). Here, I found five intronization events occurring over an evolutionary time period corresponding to 0.10 of silent site divergence (that is, the external branch leading to JEC21, since the study was only able to detect intronization events in JEC21; see Figure 1), suggesting roughly 5/0.10 = 50 intronization events per genome per unit of dS, or a per-genome rate of roughly 2.5 × 10^-6 ^to 2.5 × 10^-7 ^per genome per year. This suggests that a modern species may have experienced roughly *I *= 500 to 5000 intronization events since early eukaryotic history. Very intron-rich modern species have upwards of one hundred thousand introns [41,42], in which case intronization is unlikely to explain a large fraction of modern introns. Moderately intron-rich species have thousands or tens of thousands of introns, and appear to have lost the majority of their ancestral introns even over timescales significantly shorter than eukaryotic history (e.g., dipterans, *Schizosaccharomyces pombe*, euascomycetous fungi, *Caenorhabditis *nematodes, apicomplexans, ciliates, phytophthora; [4,6,7],43–45), suggesting high *L *values, in which case intronization may also not be a major contributor to the genomic intron complement of modern species. Intron-poor modern species have very large *L *values (greater than 99% in some species [4,6,7,43,35], in which case few introns due to intronization events are likely to remain in these species. These arguments suggest that intronization may not be a major overall contributor to modern gene structures. However, it is important to keep in mind that these estimates are necessarily quite rough.

### Quality of assembly and annotations

How robust are the present results likely to be to the limitations of current genome assemblies and annotation? There are three major potential concerns: intron prediction, sequencing errors, and genome alignments. First, the presence of cDNA transcripts reflecting that splicing event confirms that annotated 'intron' is in fact spliced, whether constitutively or alternatively. Second, sequencing errors at intron boundaries are unlikely to be a major contributor to these results, since for the vast majority of observed apparent changes either (i) there is a nearby alternative boundary, providing a simple explanation for the absence of the boundary; or (ii) at least two species lack GT..AG boundaries, which cannot be explained by rare sequence errors. Third, the low degree of divergence across these species leads to clear sequence similarity across the entire studied region: as is clear in the figures, changes in boundaries often represent single nucleotide changes within a long region of conserved sequence, allowing confidence in the true homology and quality of alignment of the studied regions overall. Notably, each of these concerns involve false positives, potentially inflating numbers of boundary changes; thus the central finding of very little change of intron boundaries is robust to such concerns.

It is also important to be clear about what sorts of changes are and are not detectable in this study. A change in intron-exon splicing boundary need not require change in GT...AG boundaries, but instead could simply be due to the emergence of a more robustly recognized boundary that overshadows the ancestral boundary. While the initial boundary movement in such a case would not be detectable in the current study, the now defunct boundary would be expected to eventually incur a mutation, which would be detected by this study. Given that for each such change both a true (unobserved) boundary change and a mutation knocking out the initial boundary are expected, the rate of mutations knocking out initial boundaries is expected to equal the true rate of boundary change, thus this scenario cannot explain the lack of observed change.

### Conservation of intron-exon structures in Cryptococcus

These results attest to the slow general rate of change of intron-exon structures in *Cryptococcus*. Previously it has been shown that intron loss and gain are very infrequent among these species [27], and that splicing boundaries of even UTR introns, the splicing of which is not required to regenerate coding sequences in transcripts, are largely conserved across the clade [29]. The present results extend this conservation to the level of conservation of the specific splicing boundaries.

Total branch length in terms of rate of silent site substitutions (dS) has previously been estimated as 0.46 across the entire clade [13], thus most neutral pairs of adjacent sites (dinucleotides) are expected to have undergone a change (specifically [1-(1-0.46)×(1-0.46)] = 0.71). That ~99.9% of boundaries are conserved thus suggests that a similar fraction are conserved by selection. Presumably, in the absence of candidate alternative boundaries, there will be strong selection for splicing, and thus to retain the single available boundary. More mysterious is selection retaining boundaries in the significant fraction of cases in which there is a nearby boundary-like motif (in particular for the simple C/TAG 3' boundary). In such cases, it could be the case that such boundary-like motifs are not able to act as alternative boundaries, leading to efficient purifying selection on the single efficient splice boundary. Alternatively, natural selection could oppose even small changes in boundary position, perhaps due to consequent indels in the encoded protein.

## Conclusion

These results show that intronization, de-intronization, and intron sliding have been rare events in the recent evolution of a genus of intron-rich fungi. Along with previous findings that intron loss and intron gain is rare among these species, this finding shows a picture of highly static intron-exon structures in *Cryptococcus*. These results suggest that genic sequence changes have not played as large a role in the evolution of intron-exon structures as previously suggested.

## Methods

I obtained genome-level alignments for regions corresponding to 28,256 coding sequence introns, previously studied by Hughes et al. [24], and identified cases where GT/C...AG splicing boundaries were not conserved at all four species at the positions corresponding to the JEC21 intron boundaries. Intron splicing was confirmed by BLAST searches of flanking exonic sequences (100 nts on each side) against 59,041 available JEC21 cDNA sequences, downloaded from NCBI, and all introns with a hit lacking the intervening sequence (i.e. ungapped) were retained. Manual inspection showed that many of the remaining cases were due to alignment ambiguities (many associated with expansion/contraction of intronic microsatellites), or to long sequencing gaps (often a long series of N's in one species, usually H99). A few other cases involved ambiguous or complex patterns involving multiple very long insertions/deletions, and were excluded. This manual filtering yielded 40 cases of non-conserved boundaries.

## Authors' contributions

SWR conceived the study, designed the experiments, performed the analyses, and wrote the manuscript. The author read and approved the final manuscript.
